# The polymorphic inheritance of *DIO2* rs225014 may predict body weight variation after Graves’ disease treatment

**DOI:** 10.20945/2359-3997000000295

**Published:** 2020-10-09

**Authors:** Ana Paula Comarella, Danilo Vilagellin, Natassia Elena Bufalo, Jessica Ferreira Euflauzino, Elisangela de Souza Teixeira, Ana Beatriz Pinotti Pedro Miklos, Roberto Bernardo dos Santos, João H. Romaldini, Laura S. Ward

**Affiliations:** 1 Universidade Estadual de Campinas Faculdade de Ciências Médicas Laboratório de Genética Molecular do Câncer Campinas SP Brasil Laboratório de Genética Molecular do Câncer, Faculdade de Ciências Médicas, Universidade Estadual de Campinas (Unicamp), Campinas, SP, Brasil; 2 Pontifícia Universidade Católica de Campinas Faculdade de Medicina Endocrinologia e Metabolismo Campinas SP Brasil Endocrinologia e Metabolismo, Faculdade de Medicina, Pontifícia Universidade Católica de Campinas (PUC-Campinas), Campinas, SP, Brasil; 3 Hospital do Servidor Público Estadual de São Paulo São Paulo SP Brasil Endocrinologia, Hospital do Servidor Público Estadual de São Paulo (IAMSPE), São Paulo, SP, Brasil

**Keywords:** Graves’ Disease, *DIO2* gene, polymorphisms

## Abstract

**Objective:**

We aimed to investigate the role of *DIO2* polymorphisms rs225014 and rs12885300 in Graves’ disease patients, mainly for controlling body weight following treatment.

**Subjects and methods:**

We genotyped 280 GD patients by the time of diagnosis and 297 healthy control individuals using a TaqMan SNP Genotyping technique. We followed up 141 patients for 18.94 ± 6.59 months after treatment.

**Results:**

There was no relationship between the investigated polymorphisms with susceptibility to GD and gain or loss of weight after GD treatment. However, the polymorphic inheritance (CC+CT genotype) of *DIO2* rs225014 was associated with a lower body weight variation after GD treatment (4.26 ± 6.25 kg) when compared to wild type TT genotype (6.34 ± 7.26 kg; p = 0.0456 adjusted for the follow-up time). This data was confirmed by a multivariate analysis (p = 0.0138) along with a longer follow-up period (p = 0.0228), older age (p = 0.0306), treatment with radioiodine (p-value = 0.0080) and polymorphic inheritance of *DIO2* rs12885300 (p = 0.0306).

**Conclusion:**

We suggest that *DIO2* rs225014 genotyping may have an auxiliary role in predicting the post-treatment weight behavior of GD patients.

## INTRODUCTION

**G** raves’ disease (GD) is a frequent autoimmune disorder affecting 20 to 50 individuals per 100,000 inhabitants ( 1 ). The production of antibodies against the TSH receptor (TRAb) promotes thyroid overactivity, which characterizes the disease ( 2 , 3 ). As a response to these elevated thyroid hormone serum levels, an increase in the basal metabolic rate ( 4 ) occurs, which results in weight loss in the majority of patients ( 5 , 6 ). In the course of treatment for the restoration of the thyroid function, an increase in body weight is expected. Great variability in the magnitude of this gain has been observed in clinical practice and has already been reported by literature ( 7 - 10 ). A neurochemical dysregulation between appetite control and body weight as the thyroid function reestablishes ( 8 , 11 ) as well as changes in basal metabolic rate as a result of the TSH fluctuations during the treatment ( 12 ) are factors that may contribute to weight gain, although there is considerable inter-individual variation ( 10 ). Searching for the factors that may influence this inter-individual variability may contribute to better patient management.

Type 2 deiodinase (D2) is involved in T4 deiodination to T3, contributing to intracellular and plasmatic T3 supply ( 13 ) and, therefore, to metabolism and energy expenditure regulation ( 14 ) in addition to the mechanisms of thermogenesis in brown adipose tissue and skeletal muscle ( 14 - 16 ). D2 is also involved in central regulation pathways that control the hypothalamic-pituitary axis and appetite ( 15 , 17 - 19 ). Type 1 deiodinase (D1) also contributes to plasmatic T3 serum levels and has a homeostatic role on the plasmatic concentrations of T4, being a drainage route in situations of T4 elevation in order to limit the substrate supply for D2 (promoting two T4 inactivations for each T3 activation) ( 20 - 22 ). D1 also promotes iodine recirculation. D1 and D2 are expressed in normal thyroid tissue and also in GD ( 23 ). The cyclic AMP (cAMP) signaling promotes enzymatic induction for D1 and D2 ( 24 ). Both genes *DIO1* and *DIO2* will undergo transcriptional regulation mediated by the high availability of intracellular T3, triggering an increased expression of D1 and reduced expression of D2 ( 13 , 25 ). Previous studies investigated the role of *DIO2* rs225014 in D2 enzyme function ( 26 - 28 ). In some of these studies, the polymorphic inheritance did not show significant differences in enzymatic biochemical properties ( 26 , 27 ). However, Castagna and cols. demonstrated that the polymorphic inheritance confers a lower efficiency of the enzyme in the conversion of T4 to T3 in muscles, decreases TSH suppression response to T4 in thyrotrophs and lowers fT3 serum levels in thyroidectomised patients ( 28 ). Also, corroborating these effects, it has been demonstrated that healthy euthyroid individuals with a polymorphic inheritance of *DIO2* rs225014 exhibit a delay in serum T3 increase after TRH stimulation ( 29 ). In addition, when evaluating active GD patients, Alina B and cols. showed that the wild type inheritance of *DIO2* rs225014 (TT) was associated with higher free T3 levels when compared to the other genotypes ( 30 ).

*DIO2* rs12885300 polymorphic inheritance has also been investigated. Peeters and cols. demonstrated that it was associated with higher D2 activity and greater availability of T3 at the tissue level, as shown by lower levels of serum T4, fT4 and rT3 and higher T3/T4 and T3/rt3 ratios ( 31 ).

We aimed to investigate the role of *DIO2* polymorphisms rs225014 and rs12885300 in susceptibility as well as the clinical features of a group of GD patients to better understand treatment impacts on body weight variations.

## SUBJECTS AND METHODS

### Patients

The Research Ethics Committee approved this retrospective study (CAAE: 39705814.0.0000.5404) that enrolled 280 GD patients living in an iodine sufficient area. The sample consisted of 233 females and 47 males, 39.63 ± 11.52 years old, and all participants provided informed consent. All patients exhibited hyperthyroidism characterized by increased fT4 and suppressed serum TSH levels, presented diffuse goiter confirmed by ultrasonography as well as increased thyroid uptake of 99mTc-pertechnetate and/or positive serum thyroid-receptor antibody (TRAb). Medical records were reviewed for information on other medical conditions and other drugs in use as well as smoking habit, body weight, height and body mass index (BMI).

We collected blood at the beginning of the treatment for serum TSH, free T4 measurement and antibodies against thyroid peroxidase (TPOAb), thyroglobulin (TgAb) and TRAb. Thyroid function was reassessed every six months thereafter. Patients who became pregnant or nursing; who were younger than 18 years old; who had any health condition (including chronic or acute diseases or surgery) that could interfere with body weight and those who used medications that could modify thyroid function or promote variation in body weight were excluded.

**Figure 1:**
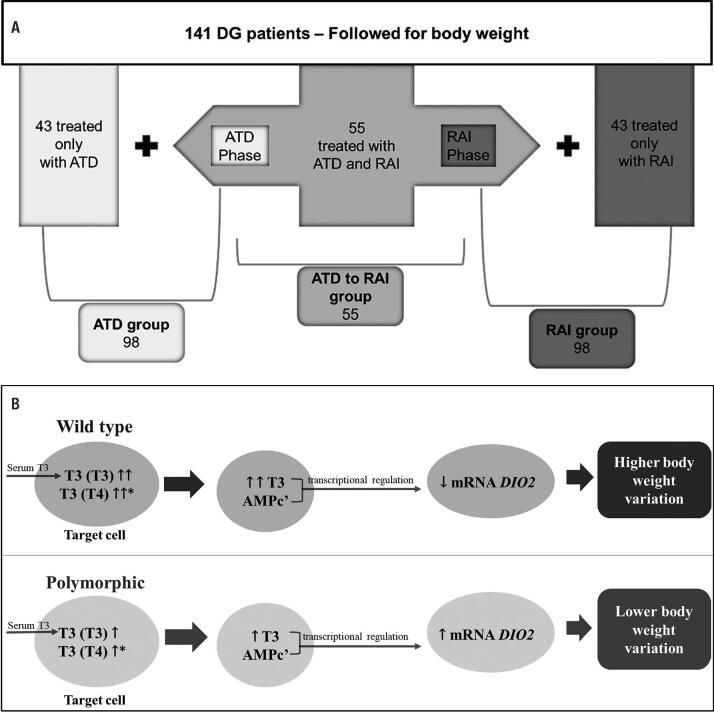
( **A** ) One hundred forty-one Graves’ Disease patients were followed for body weight. Anti-thyroid drug (ATD) group was composed by 43 patients treated only with ATD plus 55 patients initially treated with ATD. Radioiodine therapy (RAI) group was composed by 43 patients assigned to RAI (without previous ATD therapy) plus 55 patients who did not comply (relapse) with ATD administration and therefore were treated with RAI. The group ATD/RAI comprises the 55 initially treated with ATD patients and were further assigned to radioiodine therapy. ( **B** ) Hypothesis for *DIO2 rs225014* and body weight variation in Graves’ Disease. *T3 from T4 intracellular deiodination.

We were able to rigorously monitor body weight during at least one year (18.94 ± 6.59 months) in 141 GD patients. Those patients were selected after a strict protocol for body weight measurement. In each visit they used the same scale wearing no coat or shoes. Patients were divided into 3 groups according to the initial therapy offered, as summarized in Figure 1 A. A first group of 98 GD patients received an initial dose of 20 mg of Methimazole (anti-thyroid drug-ATD group) and had the following doses adjusted according to thyroid status. A second group (ATD/RAI group) comprised 55 GD patients initially treated with ATD for a period of 14.21 ± 7.83 months and were further assigned to radioiodine therapy (RAI) after which they were followed up for 30 ± 11.67 months. A third group of 98 patients received radioiodine (RAI group) and included 55 patients who did not comply with ATD administration and 43 patients who were assigned to RAI without previous ATD therapy. Body weight was analyzed according to weight (in kg) and percentage of BMI variation.

#### Patients follow-up

**Figure 2:**
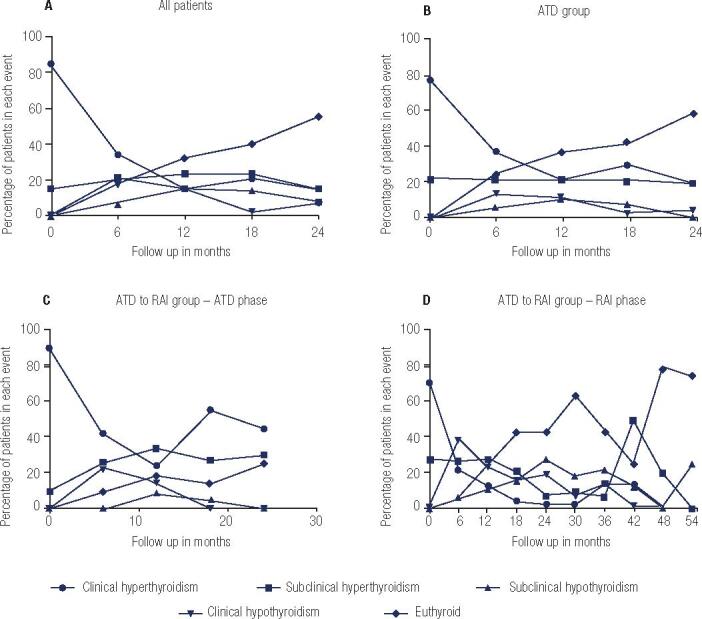
Percentage of Graves’ disease patients classified as overt hyperthyroidism, subclinical hyperthyroidism, euthyroidism, subclinical hypothyroidism and overt hypothyroidism during follow-up.

Patients were carefully examined and had their thyroid function evaluated every 6 months during a clinical outpatient visit using serum TSH and fT4 levels. In accordance with these parameters, GD patients were classified as exhibiting overt hyperthyroidism (when serum TSH levels were suppressed or were below the reference range associated with elevated fT4 serum levels); subclinical hyperthyroidism (when serum TSH levels were suppressed or were below the reference range in the presence of normal concentration of fT4); euthyroidism (when serum TSH and fT4 levels were within the reference range); subclinical hypothyroidism (when serum TSH levels were elevated in the presence of normal fT4 serum concentration) and overt hypothyroidism (when serum TSH levels were elevated and serum fT4 levels were below the reference range). The percentage of patients in each category is shown in Figure 2 .

### Controls

We included a control group of 297 healthy individuals selected from blood donors recruited at the Center of Hematology and Hemotherapy of the University of Campinas, Brazil (244 females and 53 males, 40.15 ± 11.21 years old). Control individuals were matched with patients with regard to age, gender, smoking habits and ethnicity. All control individuals presented normal TSH and fT4 serum levels. They answered an in-person questionnaire including demographic and ethnic background data, lifetime occupational history, general health conditions (i.e., dietary, physical exercises and smoking habits, drugs and medicines in use, besides previous disease information) and were submitted to a physical examination. We excluded individuals with personal or familiar history of autoimmune diseases or any suspicion of abnormality at thyroid palpation.

### Laboratory methods

The reference values for the serum levels of TSH, free T4, TgAb and TPOAb were 0.4–4.1 mIU/L, 10.3–24.4 pmol/L (0.8–1.9 ng/dL), > 40 IU/mL and > 35 IU/mL, respectively (chemiluminescent assays – DPC Immulite system, USA). The reference value for TRAb was below 10 UI/L (radio-receptor assay – RSR Ltd, UK). The thyroid volume was evaluated by ultrasound, and the reference range adopted was 6 to 15 mL. The 99mTc-pertechnetate thyroid uptake reference value ranged from 0.35 to 1.7%.

#### Identification of genotypes

Patients and controls had blood samples collected in tubes coated with EDTA (ethylenediaminetetraacetic acid). DNA was extracted from leukocytes separated from the whole blood using a standard phenol–chloroform protocol (leukocyte lysis, SDS treatment, phenol-chloroform extraction and ethanol precipitation), quantified with a UV spectrophotometer (Picodrop Limited – Cambridgeshire, UK) and stored in a freezer at -4 Fahrenheit degrees. All samples were genotyped for rs225014 and rs12885300 of the *DIO2* gene using Real-Time PCR TaqMan^®^ SNP Genotyping Assays (C_15819951_10, and C_31755153_30, respectively), employing 7500 Real Time PCR Systems (Applied Biosystems, CA), in accordance with the protocol suggested by the manufacturer. Data was analyzed for allelic discrimination using version 1.3 of the Sequence Detection software package (SDS) (Applied Biosystems, CA).

### Statistical analysis

Statistical analysis software (SAS) was used to perform the statistical analysis (SAS Institute Inc, Version 9.4, Cary, NC, USA, 2002-2008) and graphs were drawn in GraphPad Prism 6 (GraphPad Software, Inc.). Hardy Weinberg equilibrium (HWE) and Linkage disequilibrium between SNPs were performed using HAPLOVIEW software ( 32 ). Exploratory data analysis was performed through the frequency of categorical data and descriptive statistics of quantitative data. Chi-square (X^2^) and Mann-Whitney tests were used to examine the homogeneity between cases and controls regarding sex and age. Kruskal-Wallis, X^2^ and Fisher’s exact tests were used to compare the age, sex, TRAb positivity and mean serum concentrations of TSH and fT4 among the different genotypic groups. The ANOVA test was used to evaluate the relationship between the means of weight variation (kg and BMI) in the different genotypes. Simple and multiple linear regression (stepwise selection process) was used to verify the factors associated with body weight variation. The relationship between the different genotypes with the thyroid antibodies (TPOAb, TgAb and TRAb) was evaluated using the X^2^ test. The logistic regression test was used to assess susceptibility. The significance level adopted for the statistical tests was 5%.

## RESULTS

*DIO2* rs225014 and rs12885300 genotypes were in Hardy Weinberg equilibrium (p > 0.05) and no linkage disequilibrium was observed (D’<0.8). There was no difference between the total 280 GD patients and the 297 control individuals concerning the genotypic distribution of rs225014 (TT 35%, CT 47% and CC 18% *versus* TT 31%, CT 46% and CC 23%; p = 0.2846) and of rs12885300 (CC 47%, CT 45% and TT 8% *versus* CC 48%, CT 39% and TT 13%; p=0.1969), as shown in Table 1 . The genotypic distribution of the investigated polymorphisms did not differ for age, gender, positivity of antibodies (TRAb and TPOAb) and serum levels of TSH and fT4 among patient groups at diagnosis. The polymorphic inheritance of rs225014 was associated with positivity of TgAb when compared with wild type (p = 0.0209) as shown in Table 2 , that also displays other laboratory features of GD patients at diagnosis.

**Table 1:** Genotypic profile of rs225014 and rs12885300 in 280 GD patients (including 141 followed-up after introduction of ATD; ATD/RAI and RAI) and 297 healthy controls (X^2^ test). Data presented in number (and percentage)

SNPs		Controls	All GD	GD followed up for body weight	ATD	ATD/RAI	RAI
*DIO2* rs225014							
Genotypes	TT	94 (31)	98 (35)	50 (36)	40 (41)	24 (44)	37 (38)
	CT	135 (46)	132 (47)	68 (48)	42 (43)	21 (38)	41 (42)
	CC	68 (23)	50 (18)	23 (16)	16 (16)	10 (18)	20 (20)
p-value		0.1890 *		-	-	-	-
*DIO2* rs12885300							
Genotypes	CC	142 (48)	132 (47)	62 (44)	40 (41)	23 (42)	48 (49)
	CT	117 (39)	126 (45)	67 (48)	48 (49)	25 (45)	40 (41)
	TT	38 (13)	22 (08)	12 (08)	10 (10)	07 (13)	10 (10)
p-value		0.1969 **		-	-	-	-

Note: for rs225014 – TT (wild type), CT (heterozigous), CC (homozigous polymorphic) and for rs12885300 – CC (wild type), CT (heterozigous), TT (homozigous polymorphic);

* TT vs. CC genotype;

** CC vs. TT genotype.

**Table 2:** Comparison of laboratory features of *DIO2* polymorphisms rs225014 and rs12885300 at diagnosis of 280 GD patients

Laboratory features		*DIO2* rs225014				*DIO2* rs12885300			
		TT	CT	CC	p-value	CC	CT	TT	p-value
Mean ± SD	TSH	0.01 ± 0.02	0.01 ± 0.01	0.01 ± 0.01	0.1569	0.01 ± 0.05	0.01 ± 0.03	0.01 ± 0.03	0.6025
	fT4	4.12 ± 1.97	4.31 ± 3.18	5.15 ± 3.27	0.4367	3.99 ± 1.84	4.36 ± 2.95	4.89 ± 1.90	0.2016
%	TRAb (positive/negative)	29/43	51/48	20/09	0.0564	48/45	44/41	08/14	0.6780
	TPOAb (positive/negative)	34/37	47/48	19/15	0.7986	49/42	44/45	07/13	0.3418
	TgAb (positive/negative)	28/42	53/41	19/17	0.0209	48/46	44/45	08/09	0.7704

Note: p values refer to the statistical comparison of CC+TT *versus* TT.

The 141 DG patients rigorously monitored for body weight variation are described in Table 3 that includes the number of cases that presented body weight gain and the average amount of kilograms associated to each modality of treatment compared to the thyroid status evaluation.

**Table 3:** Clinical and laboratory features of all 141 GD patients monitored for body weight; after therapy with anti-thyroid drugs only (ATD); anti-thyroid drug therapy followed by radioiodine (ATD/RAI) and radioiodine therapy alone (RAI group)

Clinical/laboratory features		All patients n = 141	Groups				
			ATD n = 98		ATD/ RAI n = 55		RAI n = 98
Age (years)		39.84 ± 10.76	40.53 ± 10.70		39.70 ± 11.87		39.16 ± 11.23
Female/ male (%)		84.39/15.61	84.13/15.87		83.63/16.37		82.65/17.34
TRAb (%)	Positive	52	54		49		50
	Negative	27	30		27		20
	Missing	21	16		24		30
				**ATD**	**RAI**	**All**	
Follow-up time (months)		18.94 ± 6.59	17.76 ± 7.11	14.21 ± 7.83	30 ± 11.61	44.56 ± 11.26	30.97 ± 11.41
Free T4 (ng/dL) at diagnosis		4.87 ± 2.73	4.15 ± 2.28	4.56 ± 1.97	3.53 ± 1.94 *	4.56 ± 1.97	4.11 ± 2.06
TSH at the end of follow-up (mIU/L)		2.61 ± 3.18	0.33 ± 1.08	0.83 ± 3.43	2.98 ± 4.02	2.98 ± 4.02	3.74 ± 7.49
Free T4 at the end of follow-up (mIU/L)		2.19 ± 1.75	2.97 ± 2.01	2.98 ± 2.04	1.34 ± 0.30	1.34 ± 0.30	1.34 ± 0.31
Body weight at diagnosis (kg)		60.29 ± 11.55	60.10 ± 12.30	59.35 ± 14.05	60.93 ± 12.88	59.35 ± 14.05	61.48 ± 11.62
BMI at diagnosis		-	23.93 ± 4.01	23.7 ± 4.33	24.37 ± 4.05	23.7 ± 4.33	-
BMI at the end of follow-up		-	25.62 ± 4.37	24.78 ± 4.53	23.7 ± 4.33	23.7 ± 4.33	-
Cases with body weight gain (%)		81	79	74	85	80	83
BMI variation during the follow-up (%)		-	7.66 ± 11.87	5.49 ± 11.84	11.01 ± 9.96	16.35 ± 17.68	-
Body weight variation during the follow-up (kg)		5 ± 6.67	4.05 ± 6.27	2.73 ± 6.44	6.42 ± 5.50	8.63 ± 8.17	6.89 ± 6.57

Note:

* Measurement before radioiodine treatment.

**Figure 3:**
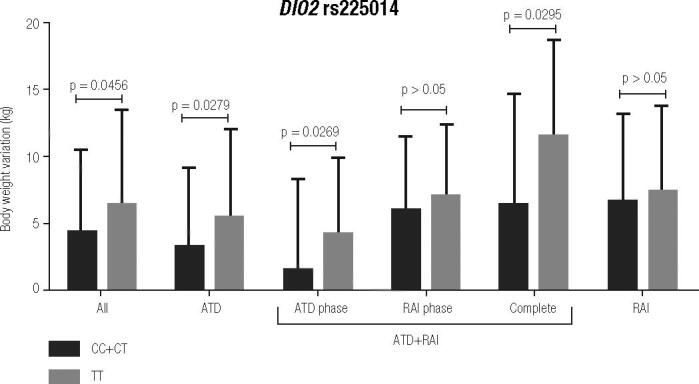
Body weight variation for *DIO2* rs225014 (weight in kg and percentage of BMI) in groups of GD patients (ANOVA, p adjusted for follow up time). All comparison TT versus CT+CC.

There was no relationship between *DIO2* rs225014 and rs12885300 genotypes with gain or loss of weight (data not show). However, we observed that the polymorphic inheritance (CC+CT genotype) of *DIO2* rs225014 was more frequent in patients with less body weight variation (4.26 ± 6.25 kg) than the wild type TT genotype (6.34 ± 7.26 kg; p = 0.0456 adjusted for the follow-up time), as shown in Figure 3 . A multivariate analysis confirmed that this polymorphism was associated with less weight variation (p=0.0138) as well as a longer follow up time (p = 0.0228), older age (p = 0.0306) and treatment with radioiodine (p = 0.0080). The lower weight variation appeared to be associated with the period of ATD therapy both in the ATD group and in the ATD/RAI group. The association was not observed during de post-RAI therapy period of the follow-up period. Unsurprisingly, patients in the ATD group spent more time exhibiting hyperthyroidism and showed lower body weight variation. In the other groups, we found no correlation between thyroid dysfunction events during the follow-up period and body weight variation.

A multiple linear regression was performed in order to evaluate the influence of thyroid status on body weight variation and confirmed the importance of *DIO2* rs225014 (TT), which can be observed in patients with longer follow-up times in the ATD/RAI group (Supplementary material – Table 1). The *DIO2* rs12885300 variant polymorphism also appeared to have a role in the prediction of body weight in the linear regression analysis of all 141 patients that were followed (p=0.0305), but this relationship was not confirmed in the treatment subgroups (Supplementary material – Table 1).

In fact, unfortunately, our power of analysis is 49.2% in the group of all patients followed for body weight (n = 141), yet 423 patients were needed in order to confirm our data.

## DISCUSSION

During the clinical course of active GD, the response to the rise of thyroid hormones is an increase in basal metabolic rate ( 4 ), which leads to weight loss in 80 to 95% of patients ( 5 , 6 ). As treatment restores the hormonal balance, weight gain is frequently observed. Previous studies reported an estimated 2.5 to 15% increase compared with the pre-morbid weight ( 7 , 8 , 33 ). In fact, 81% of our patients exhibited weight gain throughout the follow-up period, but there was a large individual variation with body weight gain from 2% to 17%.

The polymorphic inheritance of *DIO2* rs225014 has been reported to be associated with susceptibility to GD in a Russian population ( 34 ). Using a large number of patients and controls, we were not able to replicate this association in any of the *DIO2* polymorphisms evaluated, perhaps because of differences in Brazilian and Russian ethnic backgrounds. In accordance with previous reports, we found no relationship between the investigated polymorphisms with TSH ( 26 ) and fT4 serum levels ( 26 , 30 ). However, as already described, the polymorphic inheritance of *DIO2* rs225014 was associated with positivity of anti-thyroglobulin antibodies ( 35 ), which may be related to the effect of thyroid stimulation on the degree of thyroglobulin iodination ( 36 ) that results in changing its structure ( 37 ) and also by the influence of T3 levels on immune modulation and antibody production ( 38 ).

We observed that the inheritance of wild-type *DIO2* rs225014 was associated with greater variations in body weight. We may hypothesize, as represented in Figure 1 B, that under TRAb stimulation, these individuals’ availability of intracellular T3 is greater. Therefore, because the negative transcriptional factors act on the *DIO2* gene, D2 becomes less available to tissues, negatively influencing the metabolic regulation and energy expenditure in order to favor greater body weight variation. Our data, in fact, confirm higher body weight variation in patients with higher T4 serum levels at diagnosis. These findings regarding body weight variation were not confirmed in the RAI group, possibly influenced by the heterogeneity of this group, which included patients that were previously treated with MMI for several months and patients in the initial stage of GD.

Longer follow-up periods, treatment with radioiodine and older age also influenced the greater body weight variation within the multivariate analysis. The longest follow-up was previously reported as an independent risk factor for body weight gain ( 5 ), and our group also found the greatest increase in body weight in relapsed GD patients treated with RAI compared to long-term ATD-treated GD patients, perhaps because of the higher frequency of subclinical and clinical hypothyroidism events in this RAI group ( 9 ). As we advance in age, our skeletal muscle, which is an important reservoir of D2, is reduced ( 39 ). Hence, there is lower contribution of this enzyme for intracellular signaling of the thyroid hormone and metabolism, favoring body weight gain.

Our data indicate that the polymorphic inheritance of *DIO2* rs12885300, together with wild-type inheritance of *DIO2* rs225014, is associated with greater body weight variation after GD treatment, possibly due to their joint effect on T3 at the tissue level. However, other larger studies should confirm our data since the polymorphic inheritance of *DIO2* rs12885300 presented low incidence (8%) in our population, impairing the analysis of its contribution to the treatment subgroups that were evaluated.

To our knowledge, we are the first to report the association of *DIO2* polymorphisms with body weight variation following treatment of GD; nonetheless, this retrospective study has some limitations. The small sample sizes in each group of patients that were followed reduce the statistical power of our analysis, signaling the need for replication. Another important issue is the missing data associated with T3 and TRAb serum levels, which would be important to reinforce the correlations established to explain our findings on body weight variation.

We suggest that *DIO2* rs225014 inheritance may have an important auxiliary role in predicting post-treatment weight behavior in GD patients.
